# A bibliometric analysis of scientific production in the field of lingual orthodontics

**DOI:** 10.1186/s13005-019-0207-7

**Published:** 2019-09-07

**Authors:** Beatriz Tarazona-Alvarez, Rut Lucas-Dominguez, Vanessa Paredes-Gallardo, Adolfo Alonso-Arroyo, Antonio Vidal-Infer

**Affiliations:** 1 Orthodontics Teaching Unit, Department of Dental Medicine, Faculty of Medicine and Dentistry, University of Valencia, Valencia, Spain; 2 UISYS Research Unit (UV-CSIC), Department of History of Science and Information Science, School of Medicine and Dentistry, University of Valencia, Valencia, Spain; 3 Orthodontics Teaching Unit, Department of Dental Medicine, Faculty of Medicine and Dentistry, University of Valencia, C/ Gasco Oliag 1, 46010 Valencia, Spain

**Keywords:** Bracket, Bibliometric, Lingual, Orthodontics

## Abstract

**Background:**

Due to the lack of bibliometric studies in the field of lingual orthodontics in dentistry, the aim of this study was to assess the evolution and current status of activity in this field during the period 1978–2017.

**Methods:**

A bibliometric analysis of the scientific articles indexed in the Science Citation Index-Expanded of the Web of Science and in the Scopus® database was performed using the truncated terms “ling* apppli*” or “ling* orthod*” or “ling* bracket*”. The types of texts included for analysis were limited to “articles” and “reviews”. The following information was extracted from each article identified: title, authors’ name(s), institutional affiliation(s), country of origin, journal title, year of publication, type of publication, and number of citations.

**Results:**

A total of 341 articles were identified by 646 different authors, 6.2% were reviews and 93.8% were other types of journal articles. Bibliometric indicators showed a tremendous increase in the rate of publication over time with two peaks in productivity in 1989 and 2013. Fourteen authors and 15 institutional collaboration networks were identified in which European institutions were the most productive. Methodological articles were the most frequent types of research articles (28.1%), followed by case reports/series (17.1%), and narrative reviews (4.7%). Articles providing the highest quality evidence were interventional clinical trials (1.8%) and systematic reviews (0.9%). The remaining articles were non-research papers and were for information purposes only.

**Conclusions:**

Bibliometric indicators point to an irregular increase in the numbers of published works in lingual orthodontics over time. Research output is dominated by methodological articles as a technique-driven subspecialty. Although articles on lingual orthodontics are published mainly in North American journals, lingual orthodontics is largely a European domain.

## Background

The increase in demand by adults for orthodontic treatment has been mirrored by an increased availability and demand for aesthetic treatments such as lingual appliances [1]. Despite being available for over 30 years, it is perhaps only over the past decade or so that lingual therapy has entered mainstream practice and become more widely accepted as a viable treatment option for treating most malocclusions, and as a suitable alternative to conventional labial appliances [2]. Clinically improved laboratory techniques have overcome many of the difficulties that a previous generation of orthodontists encountered when they first tried to apply this innovative technique [3]. In addition to the aesthetic advantages of lingual appliances, they have also been shown to reduce the risk of enamel decalcification in comparison with labial brackets and to guarantee high precision in treatment outcomes [4].

Demand has not only increased among adult patients but since the introduction of completely customized lingual appliances, a growing number of adolescents are now being treated with lingual techniques [4].

Bibliometrics is the analysis of a set of literature to show the historical development of subject fields and patterns of authorship, publication, and use. The most common bibliometric indicators are based on the scientific productivity of researchers, organizations, and countries. This usually aims to measure the impact of research published in journals, on the basis of the number of citations a paper receives, regarded as a measure of the paper’s importance [5].

In recent years, only three bibliometric studies have been published investigating publication trends in orthodontic research [5–8] but none of them have specifically investigated the field of lingual orthodontics. The first [7] analyzed the 100 most cited articles in orthodontics from 1975 to 2011; the second [6] explored and compared the publications in three major orthodontic journals over two 5-year periods; the latest [8], identified the most cited articles from 2000 to 2015 based on the h-index. Unlike analyses of other dental specialties such as implant dentistry [9] or oral surgery [10], none of these three studies made a complete and rigorous analysis.

Given the lack of bibliometric studies of research in the specific field of lingual orthodontics, the aim of this study was to assess the evolution and current status of scientific activity in this specific field during the period 1978–2017 through a bibliometric analysis of the scientific articles indexed in the Science Citation Index-Expanded of the Web of Science (Clarivate Analytics, 1500 Spring Garden St, Philadelphia, United States) and in the Scopus® database (Elsevier B.V. Radarweg 29, 1043 NX Amsterdam, Netherlands).

## Materials and methods

### Search strategy

The search was conducted in two databases, and aimed to identify the entire body of scientific production in the field of lingual orthodontics: the Science Citation Index-Expanded of the Web of Science (SCI) and the Scopus® database. Both were selected on the basis of their broad thematic and geographic coverage of health sciences.

In the SCI database, the search was conducted employing the terms “ling* apppli*”, or “ling* orthod*”, or “ling* bracket*” in the topic field, and two inclusion criteria were applied: firstly, only documents denominated as articles or reviews were included; and secondly, and only articles categorized as Medicine, Dentistry, and Oral Surgery were included.

In the Scopus® database, the following search equation was applied containing the same terms used as the SCI: TITLE-ABS-KEY ([“ling* apppli*” OR “ling* orthod*” OR “ling* bracket*”]) AND (LIMIT-TO [DOCTYPE, “ar”]) OR (LIMIT-TO [DOCTYPE, “re”]).

The field selected in the Scopus® database (Title, Abstract, Keywords) was equivalent to the field “Topic” in the SCI. In Scopus®, the results were limited to articles and reviews, and only papers about dentistry were included. Non-English papers were excluded.

Both searches were performed in June 2018 and the period covered was defined by the earliest published research in lingual orthodontics in 1978 (first document obtained) to 2017.

The Impact Factor (IF) of each article was calculated to evaluate the impact of the journals in the SCI. The IF is calculated by dividing the number of citations in the Journal Citation Reports database year by the total number of articles published in the two previous years.

The impact of journals in Scopus® was assessed by their rank in the Scimago Journal & Country Rank (SJR) database. The SJR is calculated by indicating the average number of weighted citations received during a selected year per document published in that journal during the previous three years.

### Data collection

The following information was extracted from each article identified: title, authors’ name(s), institutional affiliation(s), country of origin, journal title, year of publication, type of publication, and number of citations.

Records were manually refined and normalized to unify terms and to remove typographical, transcription, and/or indexing errors; normalization was completed in the fields “Author,” “Organization,” and “Country of Origin.” Normalization was complicated by the numbers of different entries for a single author. In these cases, the institutional affiliations of the authors were consulted to check whether different entries belonged to the same author. If this information was not available, an Internet search was carried out to eliminate potential error. Normalization of organizations followed the same procedure. Only macro-organizations (i.e., Universities, and research centers) were included, discarding micro-organizations, such as individual departments or research units. When the same organization signed the same work more than once, it was only counted once. The “country” field was also normalized.

### Data analysis

Descriptive analysis of variables and crosstables were performed using Microsoft Access database and Excel software (One Microsoft Way Redmond, WA 98052–6399, USA).

The evolution of scientific productivity by authors, organizations, countries and journals was assessed, as well as the frequency of the appearance of keyword categories. Analysis and visualization of large networks were performed using Pajek software [11].

For the determination of the type (observational clinical trials, laboratory studies and case reports or series, reviews, interviews, systematic reviews, informative studies and others) and quality of the study, the content of the articles has been analyzed through the information available in the title and summary. The quality of the studies was assessed on the basis of methodological parameters such as the presence of a control group, the number of patients included, as well as statistical analysis.

## Results

**Fig. 1:**
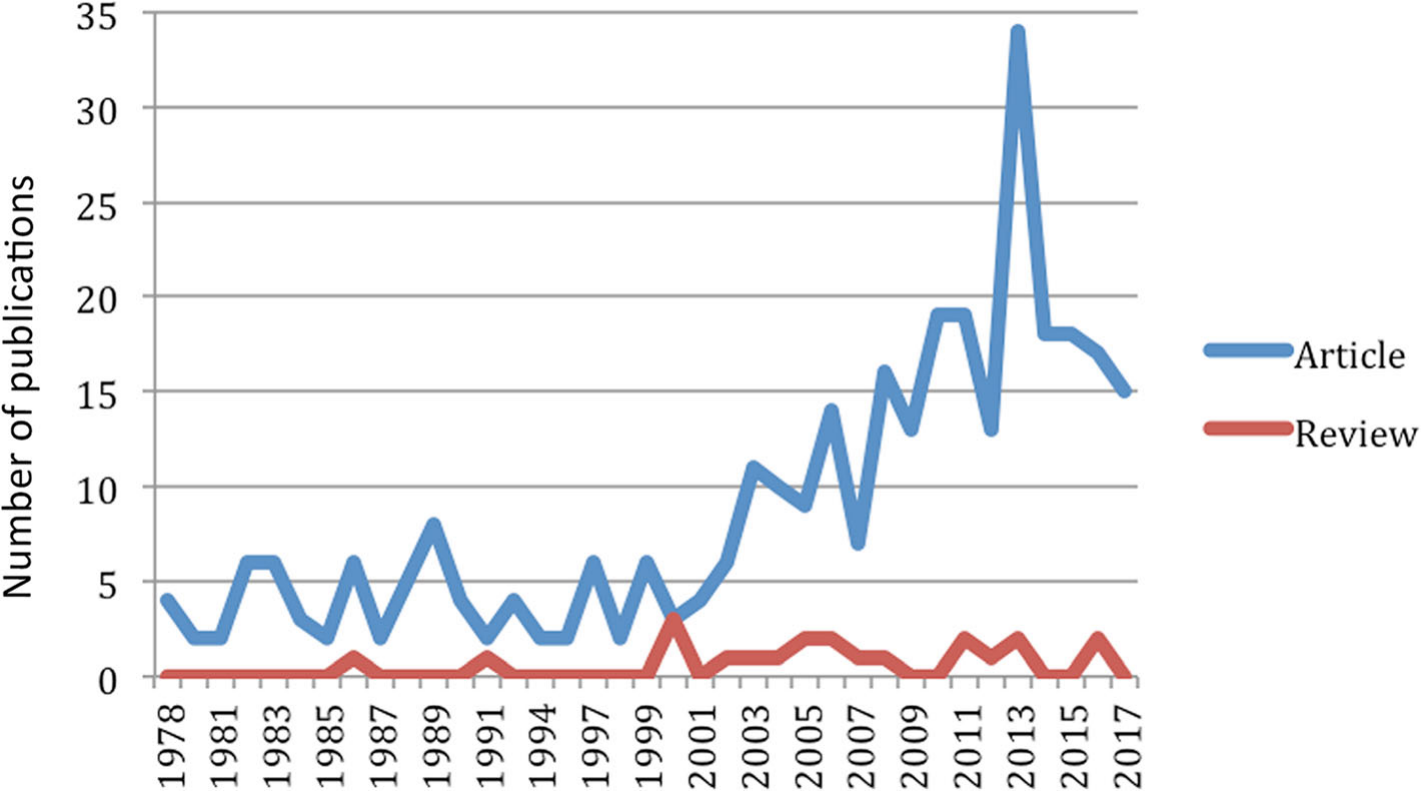
Annual evolution of scientific production from 1978 to 2018 (other types of journal articles in blue and reviews in red). The x-axis indicates year(s) of publication, and the y-axis the total numbers of publications

The annual evolution of the scientific production is shown in Fig. 1. The 40-year period 1978-2017includes the publication of 341 articles, which were placed in order according to the publication date (Fig. 1). Of 341 selected articles, 316 articles were found in Scopus®, 90 in SCI and 65 were available in both Scopus® and SCI database.

The selected 341 articles were written by 641 different authors. The average number of author per paper was 3.3. Seventy percent of authors (*n* = 452) were responsible for a single work, 17% (*n* = 109) for two works, and the remaining 13% (*n* = 85) for more than two works. Those authors with more than five publications (*n* = 17) are shown in Table 1.

**Table 1:** The most productive authors with more than five published articles appearing in the SCI and Scopus® databases

		SCI		Scopus®	
Authors	Total Articles	Articles	Cites	Articles	Cites
Wiechmann D	32	11	111	26	412
Fillion D	16	1	13	16	133
Schwestka-Polly R	14	10	97	11	90
Hohoff A	12	6	86	9	158
Geron S	11	5	55	10	107
Scuzzo G	10	1	0	10	41
Stamm T	10	5	63	8	145
Fujita K	9	1	41	8	118
Lombardo L	9	0	0	9	31
Siciliani G	9	0	0	9	31
Ehmer U	8	4	52	7	129
Hong RK	7	2	40	7	74
Bourauel C	6	5	8	5	11
Gorman JC	6	1	11	6	85
Seong-Hun K	6	3	12	4	15
Macchi A	6	0	0	6	12
Takemoto K	6	0	0	6	22

**Fig. 2:**
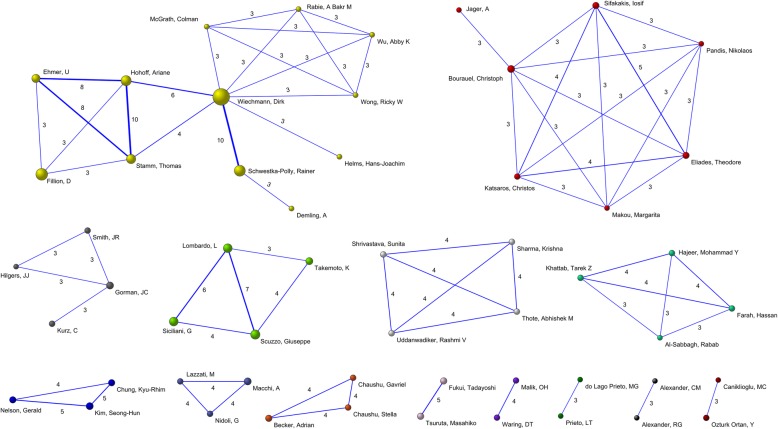
Collaboration networks between authors (defined as more than two collaborations). The size of the nodes is proportional to the number of articles published by each author. Wiechmann D leads the group with the highest level of collaboration

Based on the Pajek algorithym, 14 author collaboration networks were identified (Fig. 2). The size of the nodes is proportional to the number of articles published by each author. The largest network consists of 12 authors led by *Wiechmann D*being the most productive author with 32 publications. The same network includes three other notably productive authors: *Fillion D*, *Schwestka-Polly R* and *Hohoff A*. Each of these authors form part of the two most productive networks, linked through *Wiechmann D*. Twelve of the most productive authors belonged to collaboration networks.

The most productive institutions (*n* = 25) and their nationalities (which were the same nationalities as all the authors with more than three published works) are shown in Table 2. The *Muenster University Hospital* is in the first place with 25 articles.

**Table 2:** The most productive institutions and countries with more than three published articles appearing in the SCI and Scopus® databases

			SCI		Scopus®	
Institution	Country	Total Articles	Articles	Citations	Articles	Citations
Muenster University Hospital	Germany	25	12	138	18	357
Hannover Medical School	Germany	15	7	51	14	121
Tel Aviv University	Israel	14	7	66	11	113
Private practice Bad Essen	Germany	13	6	32	12	188
University of Ferrara	Italy	10	1	0	10	37
Kyung Hee University	South Korea	8	5	14	6	17
University of Bern	Switzerland	7	5	23	6	49
University of Bonn	Germany	7	6	17	6	16
University of Athens	Greece	6	5	11	5	12
University of Zurich	Switzerland	6	5	44	5	38
Chong-A Dental Hospital	South Korea	5	2	40	5	72
Hebrew University	Israel	5	3	13	3	22
Istanbul University	Turkey	5	2	21	5	43
University of California San Francisco	USA	5	3	12	3	11
Okayama University	Japan	4	3	32	3	36
Seoul National University	South Korea	4	4	12	4	13
Sharad Pawar Dental College	India	4	3	2	3	2
Universidade Estadual Paulista (UNESP)	Brazil	4	1	0	4	3
University of Al-Baath Dental School	Syria	4	1	12	3	16
University of Barcelona	Spain	4	1	13	4	23
University of Dusseldorf	Germany	4	2	20	2	13
University of Goettingen	Germany	4	4	10	3	5
University of Paris VII	France	4	0	0	4	98
University of Paris-Descartes	France	4	1	13	4	47
Visvesvaraya National Institute of Technology	India	4	3	2	3	2

**Fig. 3:**
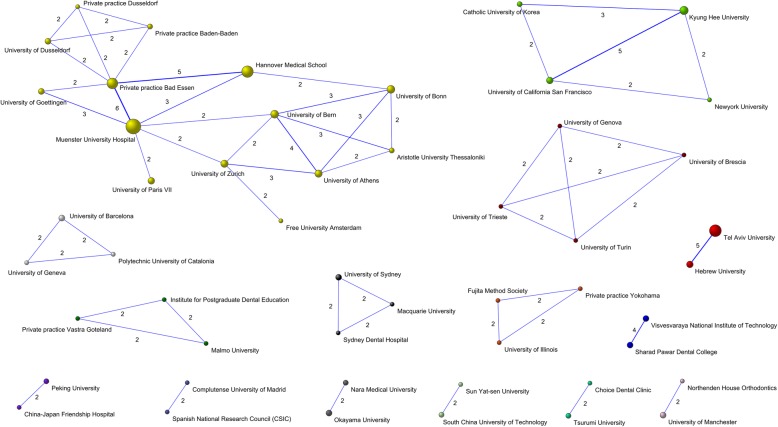
Collaboration networks between institutions (defined as more than one collaboration). The size of the nodes is proportional to the number of articles published by each institution. Muenster University Hospital is located at the core of the most important network

Collaborations between institutions (defined as more than one collaboration between institutions) obtaining a total of 15 collaboration networks are illustrated in Fig. 3. These networks are made up of 450 institutions, the most extensive being led by *Muenster University Hospital* in collaboration with 13 other institutions, most of them German institutions.

**Fig. 4:**
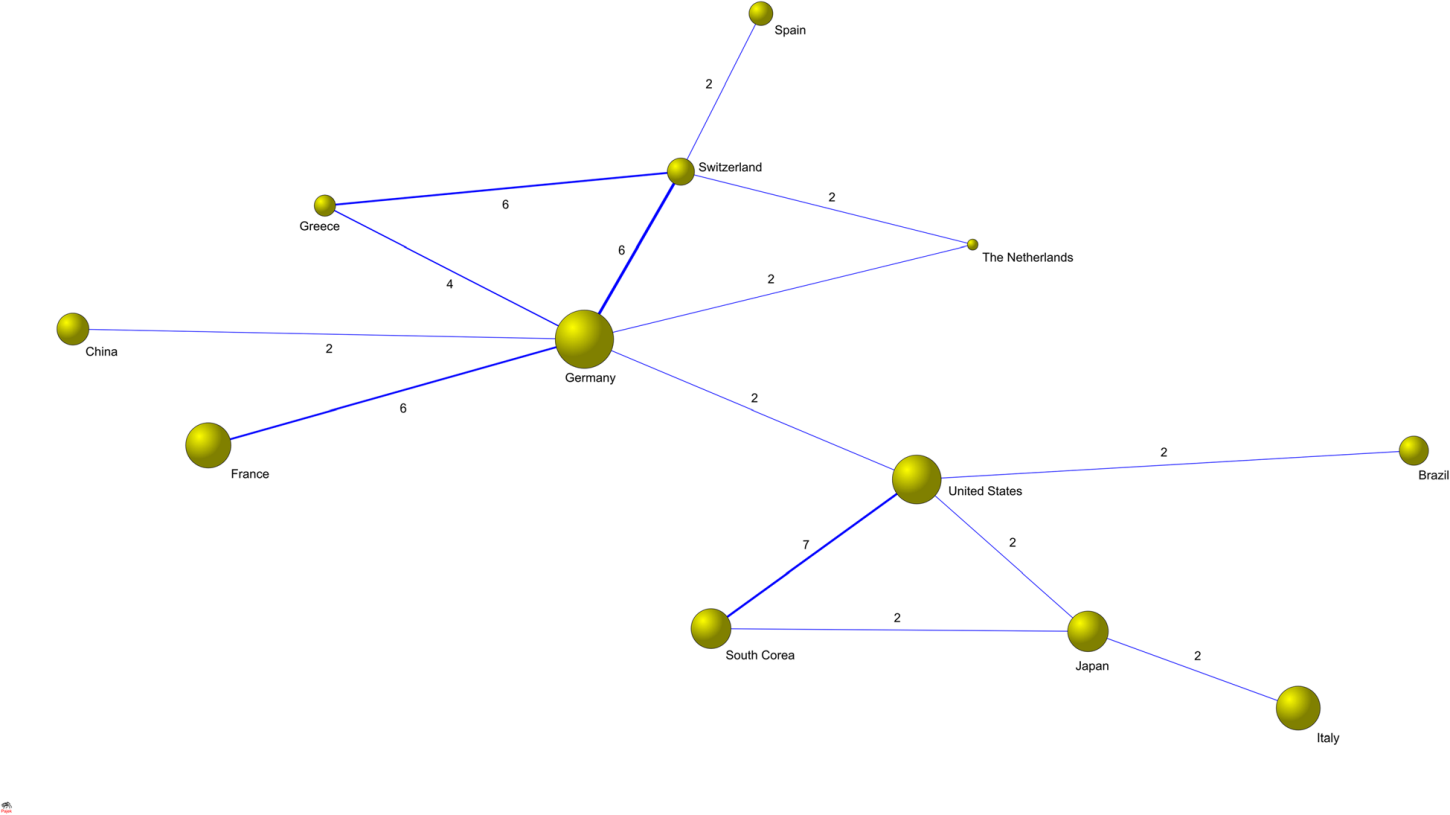
Collaboration networks between countries (defined as more than one collaboration). The size of the nodes is proportional to the number of articles published by each country. Germany followed by the USA are the central countries in the network

Regarding countries, the most productive countries with more than 20 articles published by national authors were Germany (58 articles), Italy (28 articles), South Korea (28 articles) and United States (23 articles). However, Germany and United Stateswere the two countries participating in the most collaboration works (Fig. 4).

The scientific journals with more than one work published according to the SCI and Scopus® databases are shown in Table 3. Both databases (SCI and SJR) complement one another but are not comparable since the number of journals and the means of calculating rankings are different. A distribution in classes (quartiles) may be a convenient way of comparing journal rankings across different databases (Tab. 3).

**Table 3:** Distribution of journals with more than one published article in JCR (Journal Citation Reports) and SJR (Scimago Journal & Country Rank)

	JCR category (Dentistry, Oral Surgery & Medicine)				SJR category (miscellaneous)			
Scientific Journal	Articles	Cites	IF^a^ 2016	Quartile^b^ JCR	Articles	Cites	SJR 2016	Quartile^b^ SJR
Journal of clinical Orthodontics	–	–	–	–	39	313	0.285	Q3 (1751/2864)
American Journal of Orthodontics and Dentofacial Orthopedics	19	206	1.472	Q2 (44/90)	23	358	1.265	Q1 (2/19)
International Orthodontics	–	–	–	–	20	52	0.299	Q3 (11/19)
Angle Orthodontist	18	198	1.366	Q3 (52/90)	18	256	1.216	Q1 (3/19)
								Q1 (427/2864)
Journal of Orofacial Orthopedics	–	–	–	–	16	265	0.613	Q2 (1094/2864)
								Q2 (8/19)
								Q3 (23/44)
European Journal of Orthodontics	12	65	1.622	Q2 (36/90)	13	83	1.134	Q1 (4/19)
Journal of Orthodontics	–	–	–	–	13	20	0.578	Q2 (9/19)
L’ Orthodontie francaise	–	–	–	–	13	32	0.182	Q3 (2088/2864)
Seminars in Orthodontics	–	–	–	–	10	27	0.293	Q3 (12/19)
Progress in Orthodontics	–	–	–	–	8	31	1.352	Q1 (1/19)
Korean Journal of Orthodontics	7	14	1.182	Q3 (62/90)	7	16	0.850	Q2 (7/19)
Dental Press Journal of Orthodontics	–	–	–	–	7	3	0.467	Q2 (10/19)
								Q3 (28/44)
Head and Face Medicine	6	47	1.370	Q3 (51/90)	5	24	0.584	Q2 (1175/2864)
								Q2 (50/138)
								Q3 (193/364)
								Q2 (50/110)
Journal of Oral and Maxillofacial Surgery	2	6	1.916	Q2 (31/90)	2	7	0.883	Q3 (13/44)
								Q1 (16/110)
								Q1 (95/420)
Journal of Esthetic and Restorative Dentistry	2	2	1.273	Q3 (58/90)	3	7	0.808	Q1 (24/138)

^a^*IF* Impact Factor

^b^Quartile = Distribution of the ranked journals in the JCR/SJR categories into four classes with each containing one fourth of the IF/SJR distribution of this category, whereas Q1 contains the highest and Q4 the lowest IF/SJR

- not indexed in the database

The most cited articles are shown in Table 4. Articles that have received the most citations registered in the SCI and Scopus®, referred to as “hot papers”. Kinya Fujita, inventor of the lingual bracket technique, tops the list.

**Table 4:** Articles with highest numbers of citations registered in the SCI and Scopus® database

		Cites	
Articles		SCI	Scopus®
Fujita K New orthodontic treatment with lingual bracket mushroom arch wire appliance	Am J Orthod. 1979; 76 (6): 657–675	41	66
Van der Veen MH, Attin R, Schwestka-Polly R, Wiechmann D Caries outcomes after orthodontic treatment with fixed appliances: do lingual brackets make a difference?	Eur J Oral Sci. 2010; 118 (3): 298–303	31	0
Hong RK, Heo JM, Ha YK Lever-arm and mini-implant system for anterior torque control during retraction in lingual orthodontic treatment	Angle Orthod. 2005; 75 (1): 129–141	31	38
Wiechmann D, Schwestka-Polly R, Pancherz H, Hohoff A Control of mandibular incisors with the combined Herbst and completely customized lingual appliance - a pilot study	Head Face Med. 2010;6:3.	23	0
Creekmore T Lingual orthodontics - its renaissance	Am J Orthod Dentofacial Orthop. 1989; 96 (2): 120–137	22	32
Geron S, Shpack N, Kandos S, Davidovitch M, Vardimon AD Anchorage loss - A multifactorial response	Angle Orthod. 2003; 73 (6): 730–737	21	33
Miyawaki S, Yasuhara M, Koh Y Discomfort caused by bonded lingual orthodontic appliances in adult patients as examined by retrospective questionnaire	Am J Orthod Dentofacial Orthop. 1999; 115 (1): 83–88	21	31
Geron S, Romano R, Brosh T Vertical forces in labial and lingual orthodontics applied on maxillary incisors - A theoretical approach	Angle Orthod. 2004; 74 (2): 195–201	20	–
Pauls AH Therapeutic Accuracy of Individualized Brackets in Lingual Orthodontics	J Orofac Orthop. 2010; 71 (5): 348–361	20	–
Stamm T, Hohoff A, Ehmer U A subjective comparison of two lingual bracket systems	Eur J Orthod. 2005; 27 (4): 420–426	19	–
Wiechmann D, Rummel V, Thalheim A, Simon JS, Wiechmann L Customized brackets and archwires for lingual orthodontic treatment	Am J Orthod Dentofacial Orthop. 2003; 124 (5): 593–599	–	53
Baxter J Competing discourses in the classroom: A post-structuralist discourse analysis of girls’ and boys’ speech in public contexts	Discourse Society 2002; 13 (6): 827–842	–	52
Wiechmann D A new bracket system for lingual orthodontic treatment. Part 1: Theoretical background and development	J Orofac Orthop. 2002; 63 (3): 234–245	–	40
Wiechmann D Lingual orthodontics (part 1): laboratory procedure	J Orofac Orthop. 1999; 60 (5): 371–379	–	36
Fujita K Multilingual-bracket and mushroom arch wire technique. A clinical report	Am J Orthod. 1982; 82 (2): 120–140	–	34

- not indexed in the database

A classification of the articles according to the type of study is shown in Table 5. Laboratory studies (methodological articles) are the most frequent type of research paper (28.1%), followed by case reports/series (17.1%), and narrative reviews (4.7%). The highest quality evidence in the field of lingual orthodontics is provided by interventional clinical trials and systematic reviews but these only represent a tiny proportion of all published works: 1.8 and 0.9% respectively.

**Table 5:** Articles with highest numbers of citations (hot papers) registered in the SCI and Scopus database (The empty cells mean that those articles have not received citations in that database)

Authors	Title	Journal	Cites SCI	Cites SCOPUS
Fujita, K	New orthodontic treatment with lingual bracket mushroom arch wire appliance	American Journal of Orthodontics and Dentofacial Orthopedics 1979; 76 (6): 657–675	41	66
van der Veen, MH; Attin, R; Schwestka-Polly, R; Wiechmann, D.	Caries outcomes after orthodontic treatment with fixed appliances: do lingual brackets make a difference?	European Journal of Oral Sciences 2010; 118 (3): 298–303	31	
Hong, RK.; Heo, JM.; Ha, YK.	Lever-arm and mini-implant system for anterior torque control during retraction in lingual orthodontic treatment	Angle Orthodontist 2005; 75 (1): 129–141	31	38
Wiechmann, D; Schwestka-Polly, R; Pancherz, H; Hohoff, A.	Control of mandibular incisors with the combined Herbst and completely customized lingual appliance - a pilot study	Head & Face Medicine 2010; Mar 11;6:3.	23	
Creekmore, T	Lingual orthodontics - its renaissance.	American Journal of Orthodontics and Dentofacial Orthopedics 1989; 96 (2): 120–137	22	32
Geron, S; Shpack,N; Kandos, S; Davidovitch, M; Vardimon, AD.	Anchorage loss - A multifactorial response	Angle Orthodontist 2003; 73 (6): 730–737	21	33
Miyawaki, S; Yasuhara, M; Koh,Y.	Discomfort caused by bonded lingual orthodontic appliances in adult patients as examined by retrospective questionnaire.	American Journal of Orthodontics and Dentofacial Orthopedics 1999; (): 83–88	21	31
Geron, S; Romano, R; Brosh, T.	Vertical forces in labial and lingual orthodontics applied on maxillary incisors - A theoretical approach	Angle Orthodontist 2004; 74 (2): 195–201	20	
Pauls, AH.	Therapeutic Accuracy of Individualized Brackets in Lingual Orthodontics	Journal of Orofacial Orthopedics-fortschritte Der Kieferorthopadie 2010; 71 (5): 348–361	20	
Stamm, T; Hohoff,A; Ehmer, U.	A subjective comparison of two lingual bracket systems	European Journal of Orthodontics 2005; 27 (4): 420–426	19	
Wiechmann, D; Rummel, V; Thalheim, A; Simon, JS; Wiechmann, L.	Customized brackets and archwires for lingual orthodontic treatment	American Journal of Orthodontics and Dentofacial Orthopedics 2003; 124 (5): 593–599		53
Baxter, J.	Competing discourses in the classroom: A post-structuralist discourse analysis of girls’ and boys’ speech in public contexts	Discourse & Society 2002; 13 (6): 827–842		52
Wiechmann, D.	A new bracket system for lingual orthodontic treatment. Part 1: Theoretical background and development	Journal of Orofacial Orthopedics-fortschritte Der Kieferorthopadie 2002; 63 (3): 234–245		40
Wiechmann, D.	Lingual orthodontics (part 1): laboratory procedure.	Journal of Orofacial Orthopedics-fortschritte Der Kieferorthopadie 1999; 60 (5): 371–379		36
Fujita, K.	Multilingual-bracket and mushroom arch wire technique. A clinical report	American Journal of Orthodontics 1982; 82 (2): 120–140		34

## Discussion

To address the nonexistence of bibliometric studies in the field of lingual orthodontics, the aim of this study was to assess the evolution and current status of research activity during the period 1978–2017.

The present study adopted a thorough selection process, applying several search terms (“ling* apppli*” or “ling* orthod*” or “ling* bracket*”) and detailed inclusion criteria to identify relevant articles in the SCI and Scopus® databases, which were then ranked by the number of citations received by each one.

Regarding the evolution of scientific production, some dental specialities such as Implantology [5] or Periodontics [10] have undergone increasing growth in the quantity of published research in recent years. But the field of lingual orthodontics (with 341 original studies) has followed a different pattern, with increased production up to the year 1989 followed by a fall. More recently, between 2003 and 2017, research activity has taken off again with 2013 seeing the highest number of published works ever (34 articles). This could be due to the general increased demand for orthodontic treatment by adults, the appearance on the market of new lingual orthodontic systems, and clinically improved laboratory techniques. Review articles remained constant during this period with no increase. Most of the works identified were research articles and were found in the Scopus® database.

Most of the authors published only one work. Of those with more than five published works, *Wiechmann D* was the most productive and outstanding among the other authors, with a total of 32 published works, double the number published by *Fillion D* (16 works), in second place. But on the basis of the number of citations received by each article, another different author, *Fujita K*, is notable for the 41 citations registered in the Journal Citation Report (JCR) database [12] for his 9 works.

As for the number of authors per work, the average was 3.3, a lower average than in other dental specialities such as implantology with an average of 4.66 [9] or periodontics with an average of 5.1 [13] and a much lower average than other medical fields such as cardiology with an average of 10.5 authors per work [14]. Nevertheless, the number of authors per work has increased from an average of 1.6 to 4.2 in recent years, an evolution that corresponds to other medical fields due to the multidisciplinary nature of much recent research activity [15]. One work was found to lack definite authorship, one had ten authors, and 95 works were published just by a single author.

Most institutions; 164, produced only one published work and only 10 institutions published more than five. Regarding the types of institution, most were universities and hospitals, with the exception of one private practice in *Bad Essen (Germany)* associated with the most productive author, *Wiechmann D*. European institutions – German and French – head the list, unlike other dental specialities in which most research activity takes place in North American institutions [9, 16].

The most productive authors worked in institutions in 12 different countries. The most productive countries were European, with Germany doubling Italy and South Korea in second and third place. As with the institutions publishing research, contrary to other specialities, USA is not among the three most productive nations.

All the articles included in the present analysis were published in 94 scientific journals indexed in SCI or Scopus® databases, or in both. The three scientific journals with most published works and citations registered in both databases were: *The Journal of Clinical Orthodontics, The American Journal of Orthodontics and Dentofacial Orthopedics* and *The Angle Orthodontist,* while some articles were published in scientific journals that only appeared in one database. A possible explanation is that these three scientific journals are popular, highly respected journals in orthodontics with a high impact factor that per se can influence the authors’ decision about where to publish. Another parameter to be considered is the frequency of publication of orthodontic journals (*American Journal of Orthodontics* and *Dentofacial Orthopedics*, 12 issues per year; *The Angle Orthodontist* and *The Journal of Clinical Orthodontics*, 6 issues per year), which increases the amount of content (number of articles) and boosts readership among dentistry professionals.

Not all the articles in our study were published in journals specializing in orthodontics; some were published in journals dealing with other dental specialities such as surgery or esthetic and restorative dentistry with high impact factors as noted by other authors [8].

This reflects the increasingly multidisciplinary nature of treatments involving orthodontics especially in adult patients, and the need to maximize the impact of orthodontic research.

Among the most cited articles or “hot papers,” one article by *Fujita K* [11] published in 1979 stands out with 41 citations in both the SCI and Scopus® databases, unlike other cited articles whose citations do not appear in both databases. The reason for the high number of citations may be because it was published many years ago; obviously older works may have received more citations simply because they have been available for longer. Generally, citations to papers peak in the second, third, or fourth year after publication, but some papers continue to be cited for many years. A few papers can exhibit delayed recognition. Patterns can vary depending on the type of paper, the field, and the findings reported. Papers reporting methods or techniques can gradually increase in citation frequency over several years as the methods diffuse through the community and prove their utility.

The majority of the analyzed articles were primary research articles; mainly, observational clinical studies, laboratory studies and case reports. Only a small percentage of articles corresponded to secondary research articles (reviews, systematic reviews or meta-analyses).

## Conclusions

The bibliometric indicators point to an irregular increase in the numbers of published works in lingual orthodontics over time. Research output is dominated by methodological articles as a technique-driven subspecialty. The number of articles is generally lower compared with other dental or medical specialties, which include several research fields.

## Data Availability

The datasets used and/or analyzed during the current study are available from the corresponding author upon reasonable request.
