# Correction to: A bibliometric analysis of scientific production in the field of lingual orthodontics

**DOI:** 10.1186/s13005-019-0208-6

**Published:** 2019-10-21

**Authors:** Beatriz Tarazona-Alvarez, Rut Lucas-Dominguez, Vanessa Paredes-Gallardo, Adolfo Alonso-Arroyo, Antonio Vidal-Infer

**Affiliations:** 1 Orthodontics Teaching Unit, Department of Dental Medicine, Faculty of Medicine and Dentistry, University of Valencia, Valencia, Spain; 2 UISYS Research Unit (UV-CSIC), Department of History of Science and Information Science, School of Medicine and Dentistry, University of Valencia, Valencia, Spain; 3 Orthodontics Teaching Unit, Department of Dental Medicine, Faculty of Medicine and Dentistry, University of Valencia, C/ Gasco Oliag 1, 46010 Valencia, Spain

**Correction to: Head Face Med (2019) 15:23**

**https://doi.org/10.1186/s13005-019-0207-7**

Following publication of the original article [1], the author informed that Table 5 was incorrectly captured which is actually an older version of Table 4. The corrected Table 5 is provided below.

**Table 5:** Details of the selected articles (*n* = 341) regarding the type of article and methodological parameters

Article type	n (%) *	Statistics (%)**	Control group***	Sample size	Articles
Interventional clinical trials	6 (1.8%)	6 (100%)	6 (100%)	≤ 20	1
				> 20	5
Observational studies	70 (20.5%)	50 (71.4%)	9 (12.8%)	≤ 20	8
				> 20	62
Case reports/series	58 (17.1%)	0 (0%)	–	= 1	43
				> 1	15
Laboratory studies	96 (28.1%)	0 (0%)	–	–	–
Systematic reviews	3 (0.9%)	3 (100%)	–	–	–
Narrative reviews	16 (4.7%)	2 (12.5%)	–	–	–
Interviews	2 (0.6%)	0 (0%)	–	–	–
Informative studies	40 (11.7%)	0 (0%)	–	–	–
Others	50 (14.7%)	–	–	–	–

*number of article types and percentage of all selected articles (*n* = 314)

**number of articles providing statistical analysis and percentage of all selected articles (*n* = 314)

***number of studies with control group and percentage of all selected articles (*n* = 314)

- not applicable
